# Serum Uric Acid and Disease Severity in Chronic Liver Disease: A Cross-Sectional Study of Its Association With Child-Pugh Class

**DOI:** 10.7759/cureus.116230

**Published:** 2026-09-14

**Authors:** Pratik Rathi, Vivek Pande, Fahad Idrees Shaikh, Rituraj Y Deshmukh, Arman Hussain, Zainab, Malvika Chandwani, Bhala Satish, Verbena Bezbaruah

**Affiliations:** 1 General Medicine, Narendra Kumar Prasadrao (NKP) Salve Institute of Medical Sciences and Research Center and Lata Mangeshkar Hospital, Nagpur, IND; 2 Medicine, Narendra Kumar Prasadrao (NKP) Salve Institute of Medical Sciences and Research Center and Lata Mangeshkar Hospital, Nagpur, IND; 3 Medicine, All India Institute of Medical Sciences, Madurai, IND; 4 Medicine, Dr VRK Women’s Medical College, Hyderabad, IND; 5 Medicine, Sri Aurobindo Institute of Medical Sciences, Ujjain, IND; 6 Medicine, Grant Medical College and Sir Jamshedjee Jeejeebhoy Group of Hospitals, Mumbai, IND; 7 Medicine, Tomo Riba Institute of Health and Medical Sciences, Naharlagun, IND

**Keywords:** child-pugh score, chronic liver disease, cirrhosis, cross-sectional study, hepatic encephalopathy, hyperuricemia, liver disease severity, serum uric acid

## Abstract

Background: Chronic liver disease (CLD) is a major cause of morbidity and mortality worldwide, with India accounting for the highest absolute number of cirrhosis-related deaths globally. Serum uric acid (SUA), the end product of purine metabolism, is increasingly recognised as a marker of oxidative stress and metabolic derangement and has been reported to be elevated in CLD. The Child-Pugh score remains the most widely used bedside tool for assessing hepatic functional reserve. Data regarding the association of SUA with Child-Pugh class and CLD-related complications in Indian tertiary care settings remain limited.

Objectives: The study aimed to estimate SUA levels in patients with CLD at admission, determine their association with disease severity according to Child-Pugh class, and assess their association with CLD-related complications.

Methods: This cross-sectional, observational study was conducted over 18 months in the Department of General Medicine at a tertiary care teaching hospital. Ninety-two consecutive patients with CLD aged 18-65 years were enrolled. Fasting SUA, liver function tests, haemogram, and coagulation parameters were recorded, and disease severity was graded using the Child-Pugh classification. Continuous variables were compared using the independent-samples t-test or one-way analysis of variance (ANOVA) with Bonferroni-corrected pairwise comparisons. Categorical associations were assessed using Pearson's chi-square test or Fisher's exact test, as appropriate. A two-tailed p-value <0.05 was considered statistically significant.

Results: The mean age was 47.6 years, with a male predominance (76.1%). Alcoholic liver disease was the most common aetiology (65.2%). Sixty-four (69.6%) patients had elevated SUA (>7.2 mg/dL, the cutoff applied uniformly to both sexes), with an overall mean SUA of 7.1 ± 1.9 mg/dL. Mean SUA increased progressively across Child-Pugh classes A (5.2 ± 1.1 mg/dL), B (6.9 ± 1.3 mg/dL), and C (8.4 ± 1.6 mg/dL) (ANOVA F(2,89)=37.38, p<0.001), with all pairwise comparisons significant after Bonferroni correction (all p<0.001). SUA was also significantly associated with Child-Pugh class in categorical analysis (χ²(2)=22.00, p<0.001). Patients with complications had higher mean SUA than those without complications (8.0 ± 1.5 vs. 5.6 ± 1.2 mg/dL; t(90)=8.08, p<0.001), and elevated SUA was significantly associated with any complication (χ²(1)=17.63, p<0.001). Child-Pugh class was similarly associated with complications (χ²(2)=15.68, p<0.001). Among individual complications, elevated SUA was significantly associated with hepatic encephalopathy (χ²(1)=6.15, p=0.013) but not with variceal bleeding, hepatorenal syndrome, spontaneous bacterial peritonitis, or portal hypertension (all p>0.05).

Conclusion: Hyperuricaemia was common in this CLD cohort and was significantly associated with Child-Pugh class and the overall presence of complications. These findings support a hypothesis-generating association between SUA and disease severity rather than an established prognostic or independent role. Except for hepatic encephalopathy, SUA was not significantly associated with individual complications. Larger prospective multicentric studies adjusting for renal function and other confounders are needed before SUA can be considered a clinical adjunct marker.

## Introduction

Chronic liver disease (CLD) is a progressive pathological process characterised by ongoing hepatocellular destruction and regeneration, culminating in fibrosis and cirrhosis [1]. Liver cirrhosis and other CLDs were responsible for approximately 1.47 million deaths worldwide in 2019, and India recorded the highest absolute number of deaths and disability-adjusted life-years attributable to this disease group of any single country [1]. Within the Asia-Pacific region, alcohol-related liver disease and chronic viral hepatitis B and C remain the dominant aetiologies, with non-alcoholic fatty liver disease (NAFLD) emerging as a rapidly growing contributor amid the region's changing dietary and metabolic profile [2].

Uric acid is the terminal product of purine catabolism, generated predominantly in the liver and intestine through the sequential oxidation of hypoxanthine and xanthine by xanthine oxidase. Beyond its traditional association with gout and nephrolithiasis, elevated serum uric acid (SUA) is now recognised as a marker of oxidative stress, endothelial dysfunction, and low-grade systemic inflammation -- mechanisms that are also implicated in metabolic syndrome and progressive hepatic injury [3]. In the setting of CLD, hyperuricaemia has been attributed to a combination of increased purine turnover from hepatocyte necrosis, reduced hepatic and renal clearance of urate as synthetic and excretory function deteriorates, and the metabolic derangements that accompany portal hypertension and diuretic therapy. Population-based data from the United States have shown that an elevated SUA level is independently associated with both the development of cirrhosis and the presence of elevated hepatic transaminases, even after adjustment for established risk factors [4]. A similar mechanistic link between hyperuricaemia and progressive fatty liver disease -- via insulin resistance, hepatic lipogenesis, and inflammasome activation -- has been described in patients with NAFLD [5].

The Child-Pugh score (originally the Child-Turcotte classification [6], later modified by Pugh and colleagues to replace nutritional status with prothrombin time [7]) remains the most widely used bedside tool for grading the severity of hepatic dysfunction and for predicting short- and medium-term survival in patients with cirrhosis. It combines two laboratory variables (serum bilirubin and albumin) with three clinical or coagulation variables (prothrombin time/international normalised ratio (INR), ascites, and hepatic encephalopathy) to stratify patients into Class A, B, or C, corresponding to one-year survival rates of approximately 100%, 81%, and 45%, respectively. Although newer objective scores such as the model for end-stage liver disease (MELD) have gained prominence [8], particularly for transplant allocation, the Child-Pugh score continues to be favoured in routine clinical practice for its simplicity and bedside applicability [9].

Several small, single-centre studies from South Asia have reported a positive association between SUA level and Child-Pugh class, suggesting that SUA could serve as an inexpensive, widely available adjunct to existing severity-scoring systems [10,11]. However, the strength of this association, and its relationship with specific disease complications such as hepatic encephalopathy, variceal bleeding, hepatorenal syndrome, and spontaneous bacterial peritonitis, has varied across studies and study populations, and dedicated Indian tertiary care data remain limited. Establishing this relationship more precisely could support the use of SUA as a low-cost tool for early risk stratification, particularly in resource-constrained settings where its estimation is already inexpensive and widely accessible.

The present study was therefore undertaken to estimate SUA levels at admission in patients with CLD attending a tertiary care teaching hospital, to determine the association between SUA level and disease severity as graded by the Child-Pugh score, and, as a secondary objective, to study the association between SUA level and CLD-related complications.

## Materials and methods

Study design and setting

This was a hospital-based, cross-sectional, observational study conducted in the Department of General Medicine at NKP Salve Institute of Medical Sciences and Research Center and Lata Mangeshkar Hospital, Nagpur, Maharashtra, India, from May 1, 2024, to October 31, 2025. Patients with a diagnosis of CLD were screened consecutively over a period of 18 consecutive months, as they presented to the department. The study was conducted after obtaining approval from the institutional ethics committee (IEC Reference No. 29/2024; approval date: April 18, 2024), and it was carried out in accordance with the ethical standards of the 1964 Declaration of Helsinki and its later amendments. Written informed consent was obtained from all participants before enrolment.

Sample size calculation

As no comparable prior study had reported an effect size for the primary association between SUA and Child-Pugh class that could be used for a formal a priori power calculation, the sample size was instead estimated using the anticipated proportion of raised SUA among patients with CLD reported in a prior study from a comparable tertiary care setting (anticipated proportion = 40%) [10], as a pragmatic basis for ensuring an adequately sized cohort. Using the standard formula for estimating a single proportion with a fixed absolute precision,

\[ n = \frac{Z_{1-a}^{2}\,p(1-p)}{d^{2}} \]

where Z₁₋ₐ is the standard normal deviate corresponding to a two-tailed alpha error of 5% (1.96), p is the anticipated proportion (0.40), and d is the absolute precision (±10%), the calculated minimum sample size was 92. All 92 subjects fulfilling the eligibility criteria during the study period were enrolled by consecutive sampling. This proportion-based justification, rather than a formal power calculation for the primary SUA-Child-Pugh class association, is acknowledged as a limitation of the study design (see Limitations).

Participants

Patients were enrolled consecutively, as they presented and met the following eligibility criteria. Patients aged 18-65 years of either sex with a clinical, biochemical, and/or imaging-based diagnosis of CLD, who were willing to participate, were included. Patients with a history of malignancy (leukaemia or lymphoma), gout, chronic kidney disease (defined as a pre-existing clinical diagnosis of chronic kidney disease or a documented estimated glomerular filtration rate <60 mL/min/1.73 m² for >3 months, based on history and available prior records), those on drugs known to affect serum urate (frusemide, thiazide diuretics, allopurinol, or febuxostat), those undergoing chemotherapy, those with recent major surgery or trauma, and those who did not provide informed consent were excluded. Acute kidney injury at presentation was not used as a separate exclusion criterion, other than where it manifested as hepatorenal syndrome, which was captured as a study complication; serial serum creatinine measurements were not systematically available for all participants to permit a formal, protocol-driven assessment of acute kidney injury by standard consensus criteria, and this is discussed further as a limitation.

Data collection

A detailed history (including age, sex, socio-economic status, and relevant lifestyle factors such as alcohol use) and a thorough clinical examination, including a systemic examination directed at features of CLD, were performed and recorded on a structured case record form for every participant. Relevant risk factors and comorbidities elicited on history and examination were also recorded.

Procedure

Venous blood samples were drawn after an overnight fast at the time of admission, before the initiation of any drug that could alter SUA level. Complete blood count was estimated on an automated five-part haematology analyser (Siemens Advia 2120). Fasting and post-prandial blood glucose were measured by the glucose oxidase-peroxidase method. Lipid profile was assessed on a Siemens Dimension RxL Max analyser, with total cholesterol measured by the cholesterol oxidase-peroxidase method, high-density lipoprotein cholesterol by a direct (polyethene glycol-modified enzyme) method, and triglycerides by an enzymatic endpoint method. SUA was estimated by the uricase-peroxidase (enzymatic colorimetric) method on the same Siemens Dimension RxL Max autoanalyser. A resting 12-lead electrocardiogram was recorded using a Schiller AT-2 plus machine where clinically indicated. Liver function tests (serum bilirubin, direct and indirect fractions, aspartate and alanine aminotransferases, and serum albumin) and prothrombin time/INR were estimated by standard laboratory methods on the day of admission. Disease severity was graded using the Child-Turcotte-Pugh classification, which assigns 1-3 points each for serum bilirubin, serum albumin, prothrombin time prolongation (or INR), ascites, and grade of hepatic encephalopathy; the summed score classifies patients as Class A (5-6 points), Class B (7-9 points), or Class C (10-15 points) [7]. Presence of complications was recorded on the basis of standard clinical, biochemical, endoscopic, and/or imaging criteria at the time of the index admission, applying published consensus definitions: hepatic encephalopathy was graded clinically using the West Haven criteria [12]; variceal bleed and clinically evident portal hypertension were defined by endoscopic and/or imaging evidence of oesophagogastric varices with or without active or recent bleeding [13]; hepatorenal syndrome was defined using the revised International Club of Ascites-acute kidney injury (ICA-AKI) criteria [14]; and spontaneous bacterial peritonitis was defined as an ascitic fluid absolute neutrophil count >250 cells/mm³ in the absence of a secondary source of infection [15]. Data were entered into a password-protected electronic spreadsheet, verified weekly for accuracy, and kept confidential throughout the study.

Outcome measures

The primary outcome measures were (i) the SUA level at admission, categorised as normal (≤ 7.2 mg/dL) or elevated (> 7.2 mg/dL), and its association with Child-Pugh class (A, B, or C); and (ii) the mean SUA level compared across Child-Pugh classes and between patients with and without complications. A single cutoff of 7.2 mg/dL, corresponding to the upper limit of the standard laboratory reference range in males, was applied uniformly to both sexes for categorisation, consistent with the approach used in the comparable prior studies referenced for sample size estimation and comparison [10,11]; because the standard upper reference limit is lower in females (approximately 6.0 mg/dL), this uniform threshold may under-detect hyperuricaemia in female participants, and this is discussed further as a limitation. The secondary outcome measure was the association between SUA level and the individual complications of CLD (hepatic encephalopathy, variceal bleeding, hepatorenal syndrome, spontaneous bacterial peritonitis, and portal hypertension).

Statistical analysis

Data were entered into Microsoft Excel (Microsoft Corporation, Redmond, Washington) and analysed using IBM SPSS Statistics for Windows, Version 20 (released 2011; IBM Corp., Armonk, New York). Descriptive statistics are presented as mean ± standard deviation for continuous variables and as frequency (percentage) for categorical variables. For continuous outcomes, the independent-samples Student's t-test was used to compare SUA levels between two groups (presence vs. absence of complications), and one-way analysis of variance (ANOVA) with the F-test was used to compare SUA levels across the three Child-Pugh classes; where the omnibus F-test was significant, pairwise comparisons between each pair of classes (A vs. B, A vs. C, B vs. C) were performed using pooled-variance t-tests with Bonferroni correction for three comparisons. For each categorical association, a single primary test was selected a priori based on table dimensions and expected cell counts: the Pearson chi-square test of independence (uncorrected) was used for all contingency tables in which no more than 20% of cells had an expected count of <5. Fisher's exact test was used instead wherever this assumption was violated, as recommended for sparse tables [16]. A two-tailed p-value <0.05 was considered statistically significant throughout.

Ethical declaration

The study was approved by the institutional ethics committee, and all participants provided written informed consent after being explained the purpose, procedures, and potential risks and benefits of participation. Participants were informed of their right to withdraw at any stage without any effect on their clinical care, and confidentiality of all data was maintained throughout the study.

## Results

A total of 92 patients with CLD fulfilling the eligibility criteria were enrolled consecutively over the 18-month study period. Baseline demographic and clinical characteristics are summarised in Table 1.

**Table 1:** Baseline demographic and clinical characteristics of study participants (N=92) CLD, chronic liver disease.

Characteristic	Category	n	%
Age group (years)	<30	8	8.7
	31-40	18	19.6
	41-50	26	28.3
	51-60	28	30.4
	>60	12	13.0
Sex	Male	70	76.1
	Female	22	23.9
Aetiology of CLD	Alcoholic liver disease	60	65.2
	Non-alcoholic fatty liver disease	14	15.2
	Viral hepatitis	10	10.9
	Cryptogenic	8	8.7
Child-Pugh class	Class A	22	23.9
	Class B	32	34.8
	Class C	38	41.3
Serum uric acid category	≤7.2 mg/dL	28	30.4
	>7.2 mg/dL	64	69.6

The majority of participants were middle-aged, with the highest proportion in the 51-60-year age group (30.4%) and the lowest below 30 years of age (8.7%); the mean age of the cohort was 47.6 years (calculated from grouped data). There was a marked male predominance (76.1%). Alcoholic liver disease was the leading aetiology (65.2%), followed by NAFLD (15.2%), viral hepatitis (10.9%), and cryptogenic cirrhosis (8.7%). On admission, 64 of 92 (69.6%) patients had an elevated SUA level (>7.2 mg/dL), and 70 of 92 (76.1%) patients were classified as Child-Pugh Class B or C, reflecting a cohort weighted towards moderate-to-advanced hepatic dysfunction at presentation.

Distribution of complications among study participants has been presented in Table 2.

**Table 2:** Distribution of complications among study participants (N=92)* *Percentages are of the total cohort (N=92); complications were not mutually exclusive, and some patients had more than one complication, so percentages do not sum to 100%.

Complication	n	%
Hepatic encephalopathy	30	32.6
Portal hypertension	26	28.3
Variceal bleed	20	21.7
Hepatorenal syndrome	12	13.0
Spontaneous bacterial peritonitis	8	8.7

Hepatic encephalopathy was the most frequent complication (32.6%), followed by portal hypertension (28.3%), variceal bleeding (21.7%), hepatorenal syndrome (13.0%), and spontaneous bacterial peritonitis (8.7%).

Laboratory parameters of study participants has been presented in Table 3.

**Table 3:** Laboratory parameters of study participants (N=92) Reference ranges are the standard adult laboratory reference intervals used at the study institution. Platelet count and serum albumin values were corrected from the source case-record data for internal consistency with reported units. ALT, alanine aminotransferase; AST, aspartate aminotransferase; F, female; M, male; SD, standard deviation; SGOT, serum glutamic-oxaloacetic transaminase; SGPT, serum glutamic-pyruvic transaminase.

Parameter	Mean ± SD	Reference range	Interpretation
Haemoglobin (g/dL)	10.8 ± 1.9	13.0-17.0 (M); 12.0-15.0 (F)	Mild anaemia
Total leucocyte count (cells/mm³)	9,800 ± 2,700	4,000-11,000	Near-normal
Platelet count (×10⁵/mm³)	1.85 ± 0.72	1.50-4.50	Low-normal
AST/SGOT (U/L)	86.4 ± 42.5	8-40	Elevated
ALT/SGPT (U/L)	74.2 ± 36.8	7-56	Elevated
Serum albumin (g/dL)	3.2 ± 1.5	3.5-5.0	Low-normal/low
Bilirubin, direct (mg/dL)	2.2 ± 0.8	0.0-0.3	Elevated
Bilirubin, indirect (mg/dL)	1.5 ± 0.5	0.2-0.8	Elevated
Serum uric acid (mg/dL)	7.1 ± 1.9	≤7.2 (study cutoff, both sexes)	Upper-normal/elevated

Mean haemoglobin was 10.8 ± 1.9 g/dL, consistent with mild anaemia, and the mean platelet count was at the lower end of the normal range (1.85 ± 0.72 × 10⁵/mm³). Transaminases and bilirubin were elevated, and serum albumin was reduced, in keeping with impaired synthetic and excretory hepatic function. The overall mean SUA level was 7.1 ± 1.9 mg/dL.

Comparison of mean SUA level by presence of complications and by Child-Pugh class, with post-hoc pairwise comparisons, has been presented in Table 4.

**Table 4:** Comparison of mean serum uric acid level by presence of complications and by Child-Pugh class, with post-hoc pairwise comparisons (N=92) *Independent-samples Student’s t-test (two-tailed) comparing patients with and without complications, assuming equal variances (pooled SD: 1.39). †One-way analysis of variance (ANOVA) comparing mean SUA levels across Child-Pugh classes; a significant omnibus F-test indicated a difference between at least one pair of classes. ‡Post-hoc pooled-variance t-tests based on the ANOVA mean square error, with Bonferroni adjustment for three pairwise comparisons; all pairwise differences remained statistically significant after correction. SD, standard deviation; SUA, serum uric acid.

Variable	Group	n	SUA, mg/dL (mean ± SD)	Test statistic	p-value
Complications	With complications	56	8.0 ± 1.5	t(90) = 8.08*	<0.001
	Without complications	36	5.6 ± 1.2		
Child-Pugh class	Class A	22	5.2 ± 1.1	F(2,89) = 37.38^†^	<0.001
	Class B	32	6.9 ± 1.3		
	Class C	38	8.4 ± 1.6		
Post-hoc pairwise comparisons	Class A vs. Class B	-	-	t(89) = 4.41^‡^	<0.001
	Class A vs. Class C	-	-	t(89) = 8.58^‡^	<0.001
	Class B vs. Class C	-	-	t(89) = 4.49^‡^	<0.001

Patients with complications had a significantly higher mean SUA level than those without complications (8.0 ± 1.5 vs. 5.6 ± 1.2 mg/dL; independent-samples t(90)=8.08, p<0.001). Mean SUA level also rose progressively and significantly across Child-Pugh classes, from 5.2 ± 1.1 mg/dL in Class A to 6.9 ± 1.3 mg/dL in Class B and 8.4 ± 1.6 mg/dL in Class C (one-way ANOVA, F(2,89)=37.38, p<0.001). Bonferroni-corrected post-hoc pairwise comparisons confirmed that mean SUA differed significantly between every pair of Child-Pugh classes (Class A vs. B: t(89)=4.41, p<0.001; Class A vs. C: t(89)=8.58, p<0.001; Class B vs. C: t(89)=4.49, p<0.001), indicating that the overall rise in SUA was not driven by a single class pairing but was present at each successive stage of disease severity.

Association between SUA category and Child-Pugh class has been presented in Table 5.

**Table 5:** Association between serum uric acid category and Child-Pugh class (N=92) Pearson chi-square test of independence, 3×2 contingency table (all expected cell counts ≥5) SUA, serum uric acid.

Child-Pugh class	SUA≤7.2 mg/dL	SUA>7.2 mg/dL	Total	Test statistic	p-value
Class A	15	7	22	χ²(2)=22.00	<0.001
Class B	9	23	32		
Class C	4	34	38		
Total	28	64	92		

SUA category was significantly associated with Child-Pugh class (χ²(2)=22.00, p<0.001): the proportion of patients with an elevated SUA rose from 32% (7/22) in Class A to 72% (23/32) in Class B and 89% (34/38) in Class C.

Association between SUA category and presence of any complication has been presented in Table 6.

**Table 6:** Association between serum uric acid category and presence of any complication (N=92) *Pearson chi-square test of independence, 2×2 contingency table (all expected cell counts ≥5; minimum expected count 10.96); this was pre-specified as the single primary test for this comparison. SUA, serum uric acid.

SUA category	With complications	Without complications	Total	Test statistic	p-value
≤7.2 mg/dL	8	20	28	χ²(1)=17.63*	<0.001
>7.2 mg/dL	48	16	64		
Total	56	36	92		

An elevated SUA level was significantly associated with the presence of any complication (χ²(1)=17.63, p<0.001): 48 of 64 (75%) patients with an elevated SUA had at least one complication, compared with 8 of 28 (29%) patients with a normal SUA.

Association between Child-Pugh class and presence of any complication has been presented in Table 7.

**Table 7:** Association between Child-Pugh class and presence of any complication (N=92) Pearson chi-square test of independence, 3×2 contingency table comparing Child-Pugh classes A, B, and C (all expected cell counts ≥5).

Child-Pugh class	With complications	Without complications	Total	Test statistic	p-value
Class A	6	16	22	χ²(2)=15.68	<0.001
Class B	20	12	32		
Class C	30	8	38		
Total	56	36	92		

Child-Pugh class (A, B, or C) was significantly associated with the presence of complications (χ²(2)=15.68, p<0.001): the proportion of patients with at least one complication rose from 27% (6/22) in Class A to 63% (20/32) in Class B and 79% (30/38) in Class C.

Association between SUA category and individual complications has been presented in Table 8.

**Table 8:** Association between serum uric acid category and individual complications (N=92) Each row compares the proportion of patients with the stated complication between the SUA≤7.2 mg/dL and SUA>7.2 mg/dL groups (2×2 tables). †Pearson chi-square test of independence (uncorrected), used as the pre-specified primary test where all expected cell counts were ≥5 (hepatic encephalopathy, variceal bleed, and portal hypertension). ‡Fisher's exact test, used as the pre-specified primary test for hepatorenal syndrome and spontaneous bacterial peritonitis, as >20% of expected cell counts were <5 in these 2×2 tables. A two-tailed p-value <0.05 was considered significant. SUA, serum uric acid.

Complication	SUA≤7.2 (n=28)	SUA>7.2 (n=64)	Total	Test statistic	p-value
Hepatic encephalopathy	4	26	30	χ²(1)=6.15^†^	0.013
Variceal bleed	3	17	20	χ²(1)=2.88^†^	0.090
Hepatorenal syndrome	1	11	12	Fisher's exact^‡^	0.098
Spontaneous bacterial peritonitis	1	7	8	Fisher's exact^‡^	0.427
Portal hypertension	5	21	26	χ²(1)=2.15^†^	0.143

On analysis of individual complications, an elevated SUA level remained significantly associated with hepatic encephalopathy (χ²(1)=6.15, p=0.013; 26/30 cases of hepatic encephalopathy occurred in the elevated SUA group). The associations with variceal bleed, hepatorenal syndrome, spontaneous bacterial peritonitis, and portal hypertension, considered individually, did not reach statistical significance at the 5% level, although the point estimates favoured a higher occurrence of each complication in the elevated SUA group in every instance (Table 8).

## Discussion

In this cross-sectional study of 92 patients with CLD attending a tertiary care hospital, more than two-thirds had an elevated SUA at admission, and SUA was significantly associated with both Child-Pugh class and the overall presence of complications. These findings are broadly consistent with the growing body of literature linking hyperuricaemia to the severity of CLD.

Demographic profile

The predominance of middle-aged patients (peaking in the 51- 60-year group) and of males (76.1%) in the present cohort mirrors the pattern reported by Noklang et al. in a comparable South Indian tertiary care cohort, which also observed male predominance and a similar age distribution among patients with CLD [10]. The consistency of this demographic pattern across geographically distinct Indian cohorts likely reflects the underlying epidemiology of alcohol-related liver disease, which disproportionately affects working-age men.

Aetiology and SUA level

Alcoholic liver disease was the leading aetiology in the present cohort (65.2%), a proportion broadly comparable to that reported by Vinod et al. from a tertiary centre in Agartala, where alcohol accounted for 60% of cases, followed by NAFLD, hepatitis B, hepatitis C, and autoimmune hepatitis in that series [11]. The prevalence of an elevated SUA at admission in the present study (69.6%) was also comparable to the 48% reported by Vinod et al. using a similar cutoff, and to the pattern described by Noklang et al., in whom mean SUA likewise rose with advancing Child-Pugh class [10,11]. Taken together, these findings support hyperuricaemia as a consistent biochemical accompaniment of CLD across different Indian patient populations, irrespective of modest variation in aetiological mix.

Child-Pugh class and SUA level

The stepwise rise in mean SUA level from Class A (5.2 ± 1.1 mg/dL) to Class B (6.9 ± 1.3 mg/dL) and Class C (8.4 ± 1.6 mg/dL) observed in the present study was confirmed by post-hoc pairwise testing as a significant difference at every stage. The corresponding significant categorical association between SUA level and Child-Pugh class (χ²(2)=22.00, p<0.001) closely parallels the trend described by Vinod et al., in whom mean SUA rose from 4.8 mg/dL in Class A to 5.92 mg/dL in Class B and 9.12 mg/dL in Class C [11], and by Noklang et al., who similarly reported a significant, graded rise in SUA with worsening Child-Pugh class [10]. One plausible, though unproven, biological explanation for this graded relationship is that as hepatic synthetic and excretory function deteriorate, purine turnover from ongoing hepatocellular necrosis increases. In contrast, hepatic and renal clearance of urate diminishes. It has also been hypothesised that the resulting hyperuricaemia could itself contribute to a self-perpetuating cycle of oxidative stress, endothelial dysfunction, and further hepatocellular injury [3,4]. This mechanistic pathway is drawn from prior experimental and epidemiological literature and has not been directly tested in the present cross-sectional dataset, which cannot establish a causal or temporal sequence between SUA level and hepatic dysfunction.

SUA level and complications

Patients with any complication had a significantly higher mean SUA level than those without (8.0 ± 1.5 vs. 5.6 ± 1.2 mg/dL, t(90)=8.08, p<0.001), and an elevated SUA level was significantly associated with the overall presence of complications in categorical analysis (χ²(1)=17.63, p<0.001). This is consistent with the broader literature linking hyperuricaemia to markers of hepatic decompensation and adverse outcomes in CLD [4]. However, when individual complications were examined separately, elevated SUA remained significantly associated only with hepatic encephalopathy (χ²(1)=6.15, p=0.013); associations with variceal bleeding, hepatorenal syndrome, spontaneous bacterial peritonitis, and portal hypertension did not reach statistical significance, despite point estimates in the same direction. This finding differs from some prior reports that describe significant SUA associations with each individual complication of CLD, and the discrepancy is instructive: with relatively small subgroup sizes (as few as eight patients with spontaneous bacterial peritonitis in the present cohort), individual complication-specific analyses are underpowered, and Fisher's exact test -- the statistically appropriate method for the sparse tables encountered for hepatorenal syndrome and spontaneous bacterial peritonitis [16] -- yields wider, more conservative p-values than a chi-square approximation would in a larger sample. We would caution against over-interpreting SUA as a specific predictor of any single complication of CLD on the basis of the present sample size; its association with overall complication burden and with Child-Pugh class is more consistently supported by this dataset, though still only as a cross-sectional association rather than a demonstrated predictive or causal relationship.

Laboratory correlates

The mild anaemia, elevated transaminases, and hypoalbuminaemia observed in this cohort are typical of decompensated CLD and consistent with a population weighted towards Child-Pugh Class B and C; the mean platelet count, although at the lower end of the normal range, did not, on average, indicate frank thrombocytopenia in this cohort. This laboratory profile provides internal biological context for the elevated mean SUA level (7.1 ± 1.9 mg/dL) observed, and supports the concept that hyperuricaemia in this setting may reflect, at least in part, the severity of the underlying hepatic derangement, although the cross-sectional design does not allow this to be distinguished from an isolated purine-metabolism abnormality or the influence of unmeasured confounders.

Clinical implications

SUA estimation is inexpensive and widely available even in resource-limited settings, and does not require specialised equipment beyond a standard biochemistry analyser. The consistent, graded association between SUA level and Child-Pugh class observed in the present study and in comparable Indian cohorts [10,11] is hypothesis-generating and suggests SUA merits further evaluation as a potential adjunct to established severity scores. However, this cross-sectional study did not assess whether SUA provides independent or incremental information over the Child-Pugh score or MELD after adjustment for renal function and other confounders, nor did it evaluate any prognostic or predictive value for future decompensation or mortality. Accordingly, SUA cannot at this stage be recommended as a clinical adjunct marker of disease severity, and its role, if any, as a specific predictor of individual complications requires confirmation in larger, adequately powered, prospective, multivariable-adjusted cohorts before any such recommendation could be considered.

Limitations

This study has several limitations. First, the sample size was calculated using the anticipated proportion of patients with raised SUA, as no comparable prior study provided an effect size suitable for a formal a priori power calculation for the primary SUA-Child-Pugh class association. Although the achieved sample demonstrated statistically significant associations for the primary comparisons, this proportion-based approach does not guarantee adequate power for smaller effect sizes, and the resulting complication-specific subgroup analyses, particularly for less common complications such as spontaneous bacterial peritonitis, were likely underpowered. Second, a single SUA cutoff of 7.2 mg/dL was applied uniformly to both sexes, rather than the standard sex-specific reference ranges. Because the female upper reference limit is lower (approximately 6.0 mg/dL), this approach may have under-detected hyperuricaemia among female participants and could have attenuated any sex-specific associations. Third, baseline renal function was not systematically assessed with serial serum creatinine or a formally calculated estimated glomerular filtration rate for all participants. Chronic kidney disease was excluded on the basis of history and available prior records rather than a protocol-mandated laboratory measurement, and acute kidney injury at presentation was not separately identified unless it manifested as hepatorenal syndrome. Since SUA is strongly influenced by renal function, this is an important potential confounder that could not be fully accounted for, and its omission should be considered when interpreting the observed associations. Fourth, other potential confounders of SUA level -- including diuretic exposure, precise alcohol consumption quantities, nutritional and metabolic status, and dietary purine intake -- were not adjusted for in a multivariable model. The associations reported here are therefore unadjusted and should not be interpreted as demonstrating an independent effect of SUA on disease severity. Fifth, the single-centre, cross-sectional design limits generalisability to other populations and precludes any causal or temporal inference between SUA level and disease progression. Finally, the absence of longitudinal follow-up data means that the prognostic value of SUA for future decompensation or mortality could not be assessed in this cohort. Taken together, these limitations mean the findings should be regarded as a hypothesis-generating association rather than evidence of an independent, causal, or prognostic role for SUA in CLD.

## Conclusions

In this cross-sectional study of patients with CLD, hyperuricaemia was common and was significantly associated with Child-Pugh class and with the overall presence of complications. These findings support a hypothesis-generating association between SUA and disease severity, rather than an established prognostic, causal, or independent role, given the cross-sectional design and the absence of adjustment for renal function and other confounders. Among individual complications, a statistically significant association was found only with hepatic encephalopathy; associations with variceal bleeding, hepatorenal syndrome, spontaneous bacterial peritonitis, and portal hypertension were directionally consistent but did not reach significance in this sample. Larger, prospective, multicentric studies with longitudinal follow-up and multivariable adjustment for renal function and other confounders are needed before SUA can be considered as a validated prognostic marker or an adjunct with incremental value over existing severity-scoring systems such as the Child-Pugh and MELD scores.
